# Human antibody profiling technologies for autoimmune disease

**DOI:** 10.1007/s12026-023-09362-8

**Published:** 2023-01-24

**Authors:** Lauren H. Carlton, Reuben McGregor, Nicole J. Moreland

**Affiliations:** 1 School of Medical Sciences, Faculty of Medical and Health Sciences, The University of Auckland, Auckland, New Zealand; 2 Maurice Wilkins Centre, The University of Auckland, Auckland, New Zealand

**Keywords:** Autoimmune disease, Autoantigens, Epitope spreading, Biomarker discovery, Protein array, PhIP-Seq

## Abstract

Autoimmune diseases are caused by the break-down in self-tolerance mechanisms and can result in the generation of autoantibodies specific to human antigens. Human autoantigen profiling technologies such as solid surface arrays and display technologies are powerful high-throughput technologies utilised to discover and map novel autoantigens associated with disease. This review compares human autoantigen profiling technologies including the application of these approaches in chronic and post-infectious autoimmune disease. Each technology has advantages and limitations that should be considered when designing new projects to profile autoantibodies. Recent studies that have utilised these technologies across a range of diseases have highlighted marked heterogeneity in autoantibody specificity between individuals as a frequent feature. This individual heterogeneity suggests that epitope spreading maybe an important mechanism in the pathogenesis of autoimmune disease in general and likely contributes to inflammatory tissue damage and symptoms. Studies focused on identifying autoantibody biomarkers for diagnosis should use targeted data analysis to identify the rarer public epitopes and antigens, common between individuals. Thus, utilisation of human autoantigen profiling technology, combined with different analysis approaches, can illuminate both pathogenesis and biomarker discovery.

## Introduction

Autoimmune diseases are caused by the break-down in self-tolerance mechanisms as a result of genetic and environmental influences. It is estimated that 4.5% of the global population are affected by an autoimmune disease, with the incidence increasing, especially in low and middle income countries [1]. The humoral immune response plays an important role in autoimmunity, with autoantibodies triggering downstream effects such as T cell activation and inflammation. The current diagnosis of many autoimmune diseases is lacking in sensitivity and specificity. The detection of autoantibodies is an ideal method for diagnosis as antibodies are abundant, stable, and multiple methods to detect antibodies are available. In many autoimmune diseases, such as rheumatoid arthritis, autoantibodies are circulating prior to symptom onset, allowing for early diagnosis and improved opportunities for treatment [2, 3].

There are remaining knowledge gaps as to which antigens autoantibodies recognise in many autoimmune diseases. New experimental techniques are continuously being developed with the aim of discovering novel autoantigens, including human solid surface arrays and display technologies. These new approaches have dramatically expanded the number of antigens that can be explored for involvement in autoimmune disease compared to traditional approaches. The techniques enable hundreds to thousands of human antigens to be printed on a solid surface or displayed in solution. Human samples such as serum, plasma or cerebrospinal fluid can be applied, and antibody-antigen interactions are detected. Autoantigens detected using these high-throughput and high-content approaches can then be orthogonally validated on immunoassays such as ELISA or bead-based assays such as Luminex, before being clinically validated for use as a biomarker for diagnosis. This review summarises and compares different types of human autoantigen profiling technologies, including solid surface arrays and display technologies. Recent applications for novel biomarker discovery, as well as how autoantibody profiling can contribute to improved understanding of disease pathogenesis, are highlighted.

## Solid surface arrays

Solid surface arrays display hundreds to thousands of antigens on a surface such as nitrocellulose coated glass [4]. These arrays were originally adapted from oligonucleotide arrays, which were developed in the 1990s and consisted of DNA fragments displayed on a solid surface. However, the initial oligonucleotide solid surface arrays were based on whole-genomes and included non-expressed genes that are not biologically relevant to disease. This was overcome by the advent of solid surface arrays that incorporated only proteins and peptides expressed in vivo. Solid surface arrays now provide a high-throughput method used to investigate protein-protein interactions, enzyme-substrate reactions, protein-drug interactions, and antibody-antigen interactions. Detection methods of solid surface arrays include fluorescence, mass spectrometry and surface plasmon resonance.

### Protein arrays

Protein arrays comprise full-length proteins or protein domains being displayed on the solid surface. Some of the first high content protein arrays were established in the year 2000, with over 10,000 full-length human proteins displayed on a glass slide [5]. Since then, protein arrays displaying human proteins have been used for a wide range of applications, including detecting novel antibody biomarkers in autoimmune diseases. Protein arrays can be divided into two categories based on their method of protein synthesis: those that are produced using cellular expression, or those produced using cell-free methods such as in situ synthesis. Cellular produced arrays utilise recombinant proteins expressed in a host organism prior to immobilising on the solid surface. The choice of the expression host is important to consider with common host organisms including bacteria (mostly commonly *Escherichia coli*), *Saccharomyces cerevisiae* cells (yeast), insect cells, and human cells. The expression host can affect the protein glycosylation and other post-translational modifications such as citrullination, and it is known that antibodies have the ability to recognise these post-translational modifications [3, 6]. Proteins expressed in yeast and insect cells will be glycosylated, unlike proteins expressed in bacterial hosts such as *E. coli*, though the glycosylation patterns still differ to that of humans. The number of proteins included in the array is limited to the labour and cost associated with expression and purification of the proteins. This led to the development of cell-free or in situ protein array synthesis, in which proteins are synthesised directly onto the solid surface [7]. DNA or RNA libraries are bound to the solid surface, and just hours prior to running a sample across the array, the proteins are freshly synthesised, minimising the risk of protein denaturation [7].

While custom protein arrays can be produced for a particular purpose, there are a variety of protein arrays available commercially. One of the most widely used for the discovery of novel biomarkers in autoimmune diseases is the Human ProtoArray (Thermo Fisher Scientific), based on over 9000 human proteins expressed with an N-terminal GST-tag in baculovirus, purified from SF9 insect cells and bound to a nitrocellulose-coated glass slide. The N-terminal GST tag allows for the direction of the binding of the protein on the solid surface to be controlled, which may also influence how accessible epitopes within each protein are displayed. The majority of antigens represented on the ProtoArray are metabolism-based proteins (35%), while 16% of displayed proteins are membrane associated or secreted, and 5% are nuclear based proteins. Although the Human ProtoArray was discontinued in 2018, many research groups have employed the platform to discover autoantibodies in autoimmune diseases (Table 1) including type 1 diabetes [8], systemic lupus erythematosus (SLE) [4], multiple sclerosis [9], and acute rheumatic fever [10].

**Table 1:** Description of the different human autoantigen profiling technologies discussed, including the advantages and limitations of each, and autoimmune diseases the technology has been applied to

Type of Array	Antigen Type	Array Advantages and Limitations	Array Name	Number of Antigens	Surface Type	Autoimmune Disease Application
**Protein Arrays**	Full-length proteins	**Advantages:** -Can detect autoantibodies against conformational and discontinuous epitopes. -Use of insect and yeast cells to produce antigens result in conformational epitopes and post-translational modifications to be represented. **Limitations:** -Laborious and expensive to produce antigens.	The Human ProtoArray	>9,000	Nitrocellulose treated glass slide	-Type one diabetes [8] -Parkinson’s disease ([51] -Systemic lupus erythematosus (SLE) [4] -Multiple Sclerosis [9] -Ankylosing spondylitis [52] -Sjogren’s syndrome [53] -Alzheimer’s disease [54] -Acute Rheumatic Fever [10] -Autoimmune Polyendocrine Syndrome type 1 (APS1) [48] -APECED [37] -Paediatric acute disseminated encephalomyelitis [55]
			HuProt Array	>20,000	Glass slide	-Multiple sclerosis [12] -Acute Rheumatic Fever [10] -Primary biliary cirrhosis [56] -Takayasu arteritis [57] -Multisystem inflammatory syndrome in children with a previous SARS-CoV-2 infection [36] -Bechet disease [49]
			NAPPA	80 to 12,000-	Amine coated glass slide	-Type one diabetes [15, 16] -Ankylosing spondylitis [58] -Juvenile arthritis [59] -Osteoarthritis [60] -Rheumatoid Arthritis [17]
			i-Ome Protein Array Kit	>1,600	Glass slide	-SLE [18, 19] -Rheumatoid Arthritis [20]
			ImmunoINSIGHTS	80-8,000	Microspheres	-SLE [23] -Rheumatoid arthritis [21, 22]
			In House Protein Arrays	5011	EpoxySlide	-SLE [13]
**Peptide and Protein Fragment Arrays**	Short peptides ranging 4-20 amino acids in length	**Advantages:** -Can represent the entire human proteome, providing greater breadth for autoantigen discovery. -Ideal for mapping epitopes. **Limitations:** -Conformational and discontinuous epitopes not represented. -Prokaryotic expression hosts used to produce the antigens result in post-translational modifications not represented.	The human proteome peptide array	Approx. 2.2 million	Amino functionalised microscope slides	-Multiple Sclerosis [24]
			PEPstar BioSynth	Between 50-5,000	Glass slides	-Multiple Sclerosis [23] -SLE [22]
	Longer protein fragments ranging 80-100 amino acids in length		The Human Peptide Array	Up to 42,000	Glass slides	-Multiple Sclerosis [12, 27] -Osteoarthritis [60] -Rheumatoid Arthritis [17, 30] -Sarcoidosis [31]
**PhIP-Seq**	Peptides 36 amino acids in length	**Advantages:** -Antigens are both produced and displayed by bacteriophage, reducing cost and labour. -Displays the entire human proteome including different splicing variants. -Greater throughput of samples. -Use of next-generation sequencing allows for magnitude of autoantibody response to be investigated through the number of reads detected. **Limitations:** -Lack of post-translational modifications. -Lack of complex secondary structure in the antigens displayed.	T7 Pep Library	>400,000	Bacteriophage	-Type-1 diabetes [32] -Multiple Sclerosis [32] -Rheumatoid Arthritis [32].
	Peptides 49 amino acids in length		Human PhIP-Seq Library v2	>700,000	Bacteriophage	-Paraneoplastic neurological disorders [35, 61] -APS-1 [50]
	Peptides 90 amino acids in length		-Sarcoidosis library -MIS-C library -Antygen HuScan commercial library	-1152 -250,000 >250,000	Bacteriophage	-Sarcoidosis 62] -Multisystem inflammatory syndrome in children with a previous SARS-CoV-2 infection [36]
**MIPSA**	Full-length proteins	**Advantages:** -Can detect autoantibodies against conformational and discontinuous epitopes -Greater throughput of samples -Use of next-generation sequencing allows for magnitude of autoantibody response to be investigated through the number of reads detected. Limitations: -Lack of post-translational modifications	MIPSA	>11,000	RNAClean XP beads	-Autoantibody reactivity in severe SARS-CoV-2 infections [38]
**REAP**	Extracellular and secreted proteins ranging 50-600 amino acids in length	**Advantages:** -Eukaryotic expression host results in antigen folding and post-translational modifications more like humans. -Greater through-put of samples -Use of next-generation sequencing allows for magnitude of autoantibody response to be investigated through the number of reads detected. **Limitations:** -Differing glycosylation patterns compared to humans. -Does not represent the entire human proteome.	REAP	2,688	Yeast cells	-APS-1 [40] -SLE [40] -Autoantibodies after a SARS-CoV-2 infection [41]

The more recent and most expansive protein array currently available is the HuProt Human Proteome microarray (v4.0, CDI laboratories). The 20,000 proteins cover 81% of the human proteome including 87% of predicted secreted proteins and 78% of plasma membrane proteins based on the Human Protein Atlas*.* This increased proportion of secreted and plasma membrane proteins offers an advantage compared to the Human ProtoArray for detection of novel autoantibody biomarkers that are more likely to target extracellular and exposed antigens. The HuProt Array has been employed to detect novel autoantibodies in several autoimmune diseases (Table 1) including autoimmune hepatitis [11], multiple sclerosis [12], acute rheumatic fever [10] and SLE [13].

Nucleic Acid Programmable Protein Array (NAPPA) is a popular format of in situ protein synthesis in which proteins are synthesised and captured directly onto the solid surface. For example, a NAPPA developed at the BioDesign Institute in the USA, includes over 12,000 genes encoded to express proteins with a C-terminal GST tag to ensure full translation of the protein prior to local capture on the solid surface [14]. Mammalian cell-free lysates and accessory proteins are added to synthesise the proteins in vitro enabling simple post-translational modifications to be represented such as phosphorylation and citrullination. Autoimmune diseases in which NAPPA has been utilised (Table 1) include type 1 diabetes [15, 16] and rheumatoid arthritis [17].

An emerging human protein array approach is the i-Ome Protein Array Kit (Sengenics), which comprises over 1600 antigens including signalling molecules and cytokines. The proteins are expressed in insect cells with a carboxy-biotin carrier protein signal that marks for correct protein folding to ensure display of conformational epitopes. While the inclusion of structural epitopes is an advantage of the i-Ome array, a limitation is the size, particularly as a discovery technology when the potential breadth of the autoantibody repertoire in autoimmune disease is considered. Examples of autoimmune diseases the i-Ome Protein Array Kit has been utilised to detect novel autoantibodies include SLE [18, 19] and rheumatoid arthritis [20] (Table 1). Another emerging protein array is ImmunoINSIGHTS (formally known as Serotag (Oncimmune)), which differs from the prior examples based on planar solid surfaces, as the over 8000 human proteins are immobilised on Luminex bead-based suspension arrays enabling solution phase antigen binding. Although a relatively new technology, it has been utilised (Table 1) in rheumatoid arthritis [21, 22] and SLE [23].

### Peptide and protein fragment arrays

In contrast to protein arrays, peptide arrays comprise of small protein fragments or peptides, rather than full-length proteins. Peptide arrays with overlapping, or tiled, peptides (6–20 amino acids in length) are generally used for epitope mapping within proteins that have previously been associated with autoimmune diseases [24–26]. Arrays that employ longer peptide or protein fragments (80–100 amino acids in length) are more often used to discover novel antigens associated with an autoimmune disease. Longer peptides or protein fragments are more likely to have some secondary structure and contain conformational epitopes present in native proteins, compared with short peptides [12, 27]. Like protein arrays, a limiting factor in array production is the labour and cost associated with peptide synthesis. In situ synthesis is also available for peptide arrays, in which the peptides are synthesised in parallel directly onto the solid surface [28]. Commercial peptide array synthesisers such as the *Multipep Synthetiser* (Invatis) allow for research groups to design and synthesise custom peptide arrays relevant to the autoimmune disease being studied [29].

Examples of autoimmune diseases for which peptide arrays have been utilised to map epitopes within previously identified autoantigens include multiple sclerosis [26] and SLE [25] (Table 1). The largest peptide array to date contains approximately 2.2 million overlapping 12 amino acid long peptides that represent the entire human proteome and has been used to discover novel autoantibodies in multiple sclerosis [24]. Other examples of commercially available short peptide arrays include PEPperCHIP (PEPperPRINT), BioSynth (BioSynth), and PEPstar and Pepspots (JPT Innovative Peptide Solutions), which can provide custom-designed or standard arrays based on protein sequences.

The Human Peptide Array (SciLifeLab) utilises longer peptides and protein fragments ranging 80–100 amino acids in length allowing for novel autoantigen discovery. The array is based on unique Protein Epitope Signature Tags (PrESTs) designed by the Human Protein Atlas. These PrESTs have low homology to other human protein sequences so that every fragment is unique. The 42,000 fragments, representing 94% of the human proteome, are routinely expressed in *E. coli*, purified, and immobilised on microarrays to create the Human Peptide Arrays. Examples of autoimmune diseases in which these peptide arrays have been used include multiple sclerosis [12, 27], rheumatoid arthritis [17, 30], and sarcoidosis [31] (Table 1).

## Display technologies

Display technology is a next-generation approach in which a biological organism such as bacteriophage or yeast both produces and displays the antigens. This reduces the cost of producing the array, which is often a limiting factor for solid surface arrays. Display technologies are also adaptable to a 96-well plate format allowing for higher sample throughput compared to planar solid surface arrays. Next-generation DNA sequencing of genes encoding the displayed antigens is used to detect antibody binding, in contrast to direct detection using fluorescence or similar in solid surface arrays. This provides the ability to retrieve information relating to the magnitude of the autoantibody response via the number of reads detected.

### Phage immunoprecipitation sequencing

Phage immunoprecipitation sequencing (PhIP-Seq) involves displaying a synthetic library of peptide or protein fragments of between 40 and 90 amino acids on bacteriophage. An oligonucleotide library encoding the fragments is amplified and cloned into a T7 phage display system [32, 33]. The phage library is incubated with sera, and phage bound by serum IgG is precipitated with protein A/G coated magnetic beads [33, 34]. The fragment displayed (phenotype) is linked with the fragment encoded within the genome of each phage (genotype) so that next-generation sequencing of precipitated phage provides insight into serum antigen specificity. Once a phage library has been constructed, the immunoprecipitation and sequence data can be collected within 1 week, making the technique quick and affordable [31].

The affordability and depth of data that can be obtained from PhIP-seq has led to its increasing use in autoimmune disease research (Table 1) including type-1 diabetes, multiple sclerosis, rheumatoid arthritis [32], autoimmune encephalitis [35], and most recently the post-COVID-19, multi-inflammatory syndrome in children (MIS-C) [36]. As display technologies are adaptable to a 96-well format, sample throughput is high. The depth of data obtained, as result of the incorporation of next-generation sequencing approaches, has highlighted the huge diversity of autoantibody reactivity between individuals. These unique autoantibody fingerprints maybe a hallmark of autoimmune disease that is only beginning to be appreciated [27, 37].

### Molecular indexing of proteins by self-assembly

Molecular Indexing of Proteins by Self-Assembly (MIPSA) was recently developed to complement peptide display approaches, in particular PhIP-Seq. In MIPSA over 11,000 proteins are displayed on ribosomes [38], and each protein has a unique, conjugated amplifiable DNA-barcode that enables paralleled sequencing. MIPSA was first described at the time of writing and has been used to investigate autoreactive antibodies in severe SARS-CoV-2 infections as a proof of concept [38]. As MIPSA utilises full-length antigens, this technique can be used to detect discontinuous and conformational epitopes, whereas the use of protein fragments in PhIP-Seq is more likely to detect linear epitopes [38]. The complementary nature of these approaches, including assay conditions and amplification and sequencing primers, provides opportunities for PhIP-Seq and MIPSA to be performed together in a single reaction in the future [38].

### Rapid extracellular antigen profiling

Rapid extracellular antigen profiling (REAP) was recently developed to increase the sensitivity of autoantibody detection to extracellular proteins, limited in other technologies by complex folding and post-translational modification requirements [39]. Human extracellular and secreted proteins, selected as those most accessible to autoantibodies in vivo, are displayed on the surface of yeast cells. Unlike phage produced by *E. coli*, yeast is a eukaryotic organism capable of producing post-translational modifications, although the glycosylation patterns differ slightly to that of humans [39]. Each displayed antigen is genetically barcoded and like PhIP-Seq, next-generation sequencing is used to identify autoantigens following incubation with sera and magnetic isolation of IgG bound yeast. The REAP library consists of 2688 extracellular proteins, encompassing a wide range of protein families and representing 87% of all human exoproteins. The length of the extracellular regions displayed ranges between 50 and 600 amino acids [40]. REAP is a very new technique, and proof of concept was shown in the autoimmune diseases APS-1 and SLE [40] (Table 1). Of note, the technology was also utilised to show that patients with severe SARS-CoV-2 infections develop autoantibodies, indicative of immune dysregulation [41]. The increased sensitivity for REAP to identify extracellular autoantigens was illustrated by the identification of autoantibodies to chemokines, cytokines, and growth factors in SLE patients [40]. This adds to the autoantibodies to intranuclear proteins identified with the Human Protoarray [4] and provides new avenues for research into SLE pathogenesis.

## Strengths and limitations of human antigen profiling technologies

Each of the solid surface array and display technologies has advantages and limitations, and the selection of technology should be driven by the projects main purpose. Short peptide antigens utilised in many of the array technologies are limited in secondary structure but maybe well suited if the primary goal is epitope mapping. Synthetic peptides and those generated using prokaryote expression systems, including PhIP-Seq [32], will lack post-translational modification but offer the advantage of an increased coverage of the human proteome, which may be desirable when the autoantigen repertoire is largely unknown. In contrast, full-length protein arrays, MIPSA and REAP that employ eukaryotic expression hosts are more likely to present conformational and post-translationally modified epitopes. This is important as it is estimated that 90% of autoantibodies recognise conformational epitopes [26, 42] and modifications such as citrullination [3].

These considerations are exemplified by the fact that the various technologies have detected different, and often non-overlapping autoantigens, in the same disease. For example, PhIP-Seq did not detect the known autoantigen insulin in type-1 diabetes, or citrullinated proteins associated with rheumatoid arthritis [32], likely due to the lack of conformational epitopes and post-translational modifications represented. However, PhIP-Seq did detect two clusters of peptides that clearly distinguished rheumatoid arthritis patients from healthy controls as well as a new epitope in multiple sclerosis patients [32]. Peptide approaches can also identify epitopes that are exposed during the disease process, such as during inflammatory induced tissue damage, that are not able to be detected using technology based on full-length proteins. For example, a peptide approach identified epitopes in myelin oligodendrocyte glycoprotein (MOG) and myelin basic protein (MBP) in patients with multiple sclerosis proteins [26], but these proteins were not identified using the Human ProtoArray [9]. Similarly, there was little overlap in the autoantigens identified in multiple sclerosis patients in a side-by-side comparison of the Human Peptide Array and the HuProt Array [12]. This highlights a potential need to consider multiple profiling technologies to ensure all possible autoantigen sequence space is covered for a particular disease.

## Data analysis approaches

The data generated from the different technologies is platform specific, which influences downstream analysis. Array technology tends to generate fluorescence data with higher fluorescence intensities equating to increased binding of autoantibodies to immobilised antigens, whereas display technologies generate sequencing count data with higher read counts equating to more binding to the antigen displayed. Prior to further data analysis, raw data is generally pre-processed and normalised in a platform specific manner to generate processed, continuous data for input into various analysis pipelines.

**Fig. 1:**
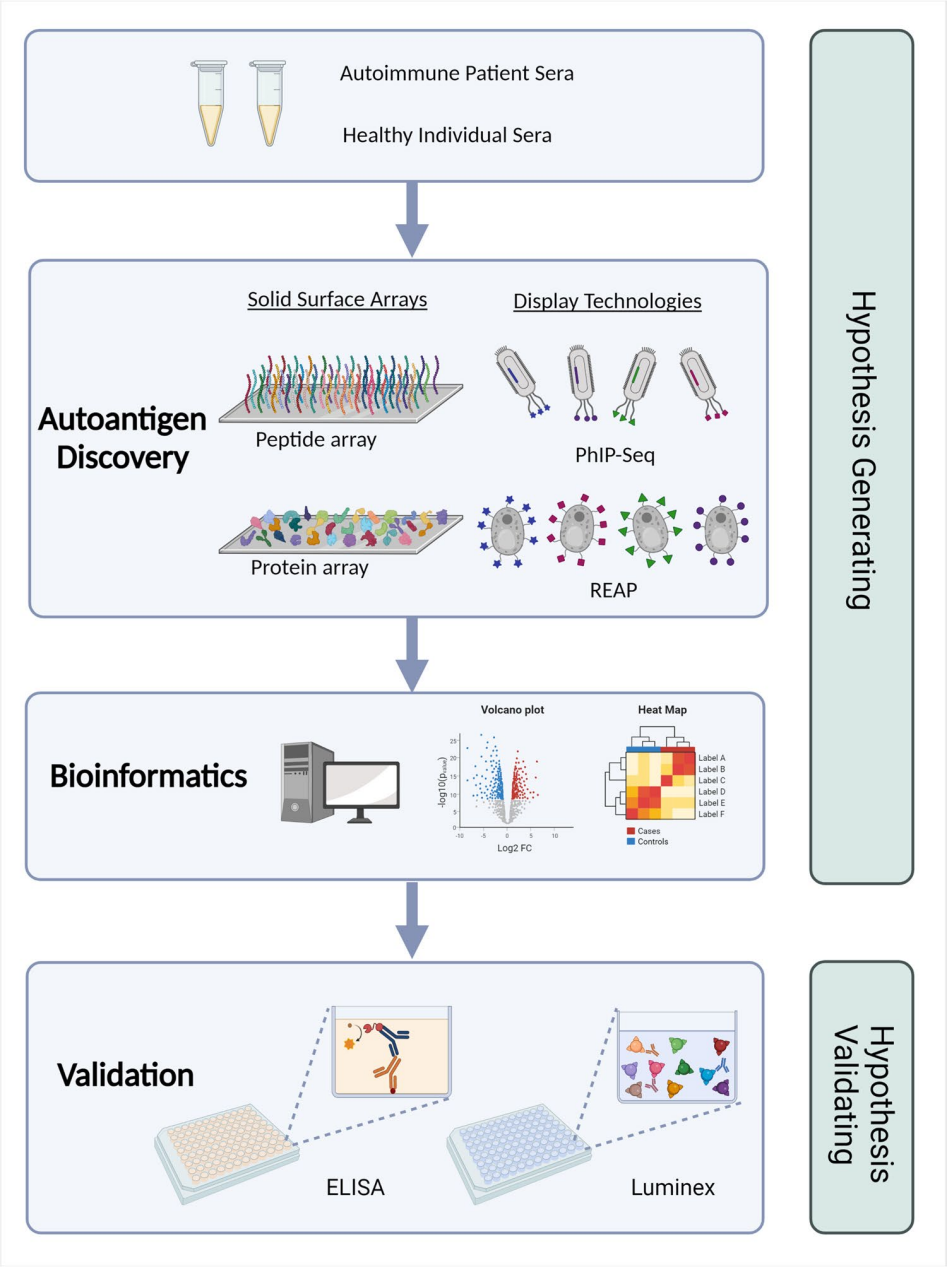
Flow chart depicting the process of autoantigen discovery using human autoantigen profiling technologies. Autoimmune patient and healthy control sera are used in the autoantigen profiling technology in the Autoantigen Discovery phase. Complex bioinformatics is required, including targeted data analysis to filter for public epitopes and antigens for novel biomarker discovery. Detected antigens are then orthogonally validated on an immunoassay such as ELISA or Luminex bead-based assays with a larger sample cohort to confirm novel findings and inform new insights into disease pathogenesis. Figure created with Biorender.com

A common approach to identify disease specific autoantibodies with biomarker potential is to compare reactivities in serum from the disease group to that of control groups. The control group maybe drawn from a healthy population, but wherever possible, these comparator groups should be closely matched with the disease group to minimise confounders and enable identification of true, disease-specific markers. This type of analysis generates fold-change and statistical *P* values (often corrected for multiple comparisons and reported as a false-discovery-rate (FDR) or *q* value), which can be used as cut-offs to narrow down the potential autoantigens to those with the largest and most significant fold-change compared to controls. These results are often visualised as volcano-plots (Fig. 1) and the identified autoantigens referred to as “hits” [10, 43]. An alternative approach is to use hierarchical clustering techniques to identify autoantigens that cluster cases from controls, often visualised as heatmaps with dendrograms (Fig. 1) [10, 40]. These two analyses can also be combined, with only the enriched, significant hits being subjected to hierarchical clustering to then identify potential autoantigens that segregate disease groups from controls. More recently, as datasets have increased in complexity, including the need to compare multiple disease groups across different platforms, more powerful multivariate analyses have become necessary. These include principal component analysis (PCA) to visualise large amounts of data from multiple groups, as well as partial-least-squares discriminant analysis (PLS-DA) to identify hits that drive the discrimination between groups [44].

Potential autoantigens that have been identified statistically can also be interrogated with respect to the pathophysiology of disease to further narrow down the hits for validation. For example, hits can be subjected to pathway analysis to identify autoantigens from relevant disease pathways [45] or filtered by expression location to identify those found in disease relevant tissues [46]. Hits from different platforms can also be compared allowing for identification of autoantigens detected by multiple platforms, which may indicate more robust targets [10]. Finally, data can be converted into binary values based on a specific cut-off, classifying each sample as reactive or non-reactive to a potential autoantigen. Binary data can provide further insight into the diagnostic potential of a hit, with the “cut-off” value generally a trade-off between sensitivity and specificity, assessed using receiver operator curve (ROC) analysis [47]. Ultimately, the statistical approach applied in any autoantibody profiling project should be that most suited to the characteristics and distribution of the dataset, as well as the hypothesis being explored.

## Validation and disease insights

The autoantigens identified from the approaches described tend to be hypothesis generating, and it is recommended that the targets identified are orthogonally validated using immunoassay methods such as ELISA and Luminex bead-based assays (Fig. 1). Discovered targets can be coated onto an ELISA plate or coupled to beads to generate a specific autoantigen assay on which a larger, or different cohort of the same disease can be assessed to confirm novel findings and inform new insights into pathogenesis [10, 24, 25, 27, 30, 48–50]. Several representations of an antigen can be included such as short peptides, protein fragments, or full-length proteins to maximise the antigen-epitope space investigated. The antibody isotype investigated in the autoantigen array is also an important consideration. Most studies focus on IgG as this is the most stable and abundant isotype; however, detection of IgA in rheumatoid arthritis [30] and MIS-C [36] provided additional insight into autoantibodies associated with mucosal surfaces in these diseases.

Increasingly, studies using human antigen profiling technologies are recognising the substantial heterogeneity of autoantibody reactivities among individuals, with the majority being private epitopes (found in few individuals) compared to public epitopes (found in many individuals) [25–27, 32, 37]. For example, a study of 92 multiple sclerosis patients using a Human Peptide Array found 64% of the antigens were reactive in only one individual [27], and marked diversity in autoantigen reactivity was observed in patients with SLE using REAP technology [39]. This heterogeneous autoantibody profile suggests epitope spreading, that is the presence of autoantibodies beyond those that trigger disease, may be an important mechanism in the progression of many autoimmune diseases, contributing to inflammatory tissue damage and disease symptoms. It follows that for studies focused on identifying autoantibody biomarkers for diagnosis, data analysis should filter for the rarer public epitopes and antigens, common between individuals with the same disease (or disease subgroup).

In summary, human solid surface arrays and display technologies are powerful high-throughput approaches capable of identifying novel autoantigens with biomarker potential, as well as illuminating the pathogenesis of autoimmune diseases. The human autoantibody profiling technology field is rapidly changing, as shown by the discontinuation and emergence of technologies throughout the past two decades. There are advantages and limitations to the various platforms, and multiple approaches should be employed to capture the full diversity of autoantibodies in a particular disease. This includes not only chronic autoimmune disease, but also post-infectious sequelae such as acute rheumatic fever, and those associated with SARS-CoV-2 infection, that have an ever-growing global burden as a result of the COVID-19 pandemic.
